# Kai-Bi-Bu-Fei Decoction Protects Mice Against Influenza Virus-Induced Severe Pneumonia via Gut Microbiota–Short Chain Fatty Acid Axis

**DOI:** 10.3390/ph19071029

**Published:** 2026-06-30

**Authors:** Mingzhe Wang, Bei Xue, Herong Cui, Miao Cheng, Jintong Li, Zhihong Ren, Tianzhen Liang, Weicheng Nie, Liqiong Song, Chengjun Ban

**Affiliations:** 1 Respiratory Department, Dongzhimen Hospital, Beijing University of Chinese Medicine, Beijing 100700, China; wmz0723@foxmail.com (M.W.); xuebei@bucm.edu.cn (B.X.); chengxinxin321@126.com (M.C.); bucmlijintong@163.com (J.L.); 20230941328@bucm.edu.cn (W.N.); 2 School of Chinese Materia Medica, Beijing University of Chinese Medicine, Beijing 102488, China; herongcui@outlook.com (H.C.); polarisa1225@163.com (T.L.); 3 National Key Laboratory of Intelligent Tracking and Forecasting for Infectious Diseases, National Institute for Communicable Disease Control and Prevention, Chinese Center for Disease Control and Prevention, Beijing 102206, China; renzhihong@icdc.cn

**Keywords:** Kai-Bi-Bu-Fei Decoction (KBD), influenza virus-induced severe pneumonia, gut microbiota, short-chain fatty acids, fecal microbiota transplantation

## Abstract

**Background**: Kai-Bi-Bu-Fei Decoction (KBD) is derived from the canonical Traditional Chinese Medicine formulas Xuan-Bai-Cheng-Qi and Ma-Xing-Shi-Gan. It has been employed for decades in the treatment of severe pneumonia with significant clinical efficacy. This study aimed to evaluate the protective effects of KBD against influenza virus-induced severe pneumonia in a murine model and to elucidate the underlying molecular mechanisms. **Methods**: The chemical profile of KBD was characterized using UPLC-Q-TOF-MS. A severe pneumonia model was established in C57BL/6J mice via intranasal infection with influenza A/Puerto Rico/8/34 (H1N1, PR8). Multiple parameters, including 14-day survival rate, body weight, lung index, histopathological changes, viral load, and pulmonary cytokine/chemokine levels, were assessed. Furthermore, multi-omics analyses were integrated to characterize the gut microbiota and metabolic profiles. Fecal microbiota transplantation (FMT) was subsequently performed to validate the functional role of the gut microbiota and its metabolites. **Results**: KBD treatment significantly improved the survival rate by 40%, reduced the lung index by 27.85%, and alleviated lung injury. It also markedly lowered the viral load by 80.88%, suppressed pro-inflammatory cytokine levels, and restored intestinal barrier integrity. Mechanistically, KBD restored gut microbiota diversity by increasing the abundance of *Firmicutes* and *Bacteroidetes*, enriching beneficial genera such as *Bifidobacterium* and *Faecalibaculum*, and reducing *Verrucomicrobiota*. Integrated transcriptomic and metabolomic analyses revealed that KBD enhanced short-chain fatty acid (SCFA) metabolism and up-regulated pyruvate metabolism. Finally, FMT confirmed that the therapeutic benefits of KBD were transferable via the microbiota to microbiota-depleted mice. **Conclusions**: KBD exerts robust protection against severe influenza pneumonia, a process primarily mediated by the gut microbiota–SCFA axis. The enhancement of mitochondrial energy metabolism also appears to play a critical role in its therapeutic mechanism.

## 1. Introduction

Influenza virus-induced pneumonia remains a major global health threat, accounting for an estimated 1 billion infections each year, 3–5 million cases of severe illness, and approximately 500,000 deaths worldwide [1,2,3]. Neuraminidase inhibitors constitute the primary antiviral therapy, yet their clinical effectiveness is increasingly challenged by rapid antigenic drift, the emergence of drug-resistant strains, and the narrow therapeutic window for timely administration [4,5]. Beyond incomplete pathogen control, severe disease is frequently driven by dysregulated host responses, including excessive inflammation and secondary tissue damage. Consequently, alternative treatment strategies were warranted that can strengthen antiviral immunity, mitigate immunopathology, and improve outcomes in influenza virus-induced severe pneumonia. Traditional Chinese Medicine (TCM) has long been used for respiratory infections in China and is receiving growing international attention as a complementary approach. Accumulating clinical and experimental evidence indicates that multi-herb formulas can exert multi-target, multi-pathway regulatory effects, including attenuation of pulmonary inflammation, relief of respiratory symptoms, and facilitation of recovery in influenza virus-induced severe pneumonia [6,7,8]. *Kai-Bi-Bu-Fei Decoction* (KBD) is a classical empirical prescription derived from two foundational formulas: *Xuan-Bai-Cheng-Qi Decoction* and *Ma-Xing-Shi-Gan Decoction. Xuan-Bai-Cheng-Qi Decoction* was recorded in “Systematic Differentiation of Warm Diseases” by Wu Jutong, a renowned physician of the Qing Dynasty. It is composed of *Gypsum Fibrosum*, *Rheum officinale* Baill., *Prunus armeniaca* L., and *Trichosanthes kirilowii* Maxim. *Ma-Xing-Shi-Gan Decoction* originates from the “Treatise on Febrile Diseases” by Zhang Zhongjing (c. 150–219 AD). It consists of four herbs: *Ephedra sinica* Stapf, *Prunus armeniaca* L., *Glycyrrhiza glabra* L., and *Gypsum Fibrosum*. Clinical observations from domestic centers suggest that KBD may alleviate cough, sputum production, and gastrointestinal symptoms (e.g., abdominal distension) in patients with influenza virus-induced severe pneumonia, and may reduce mortality risk in severe cases by restoring immune homeostasis [9,10,11]. However, despite decades of clinical practice, the mechanisms underlying KBD activity remain insufficiently clear, which limited its integration into contemporary pharmacology and evidence-based practice.

In parallel, the gut–lung axis has emerged as a major frontier in research on respiratory viral infections. Studies in both China and abroad have shown that influenza A virus (IAV; hereafter referred to as IFV) infection can profoundly reshape gut microbiota (GM) composition, reduce the abundance of beneficial taxa, alter the production of key microbial metabolites such as short-chain fatty acids (SCFAs), and compromise intestinal barrier integrity [2,12,13,14,15]. This dysbiotic state may amplify pulmonary injury through immune–inflammatory cascades, whereas a balanced GM and its metabolites can support antiviral defense by modulating immune cell function, restraining excessive inflammation, and maintaining both intestinal and pulmonary mucosal barriers [2,16,17,18]. These insights have catalyzed growing interest in microbiome-targeted interventions and “postbiotic” strategies centered on microbial metabolites and barrier repair. Notably, the gut–lung axis framework aligns with the classical TCM concept that “the Lung and Large Intestine are internally–externally related,” offering a contemporary biological lens through which to interpret gut-directed components of TCM prescriptions. Within this context, KBD is particularly relevant because its formulation rationale emphasizes coordinated regulation of the lung and large intestine. A central mechanistic question therefore remains: Does KBD alleviate influenza virus-induced severe pneumonia by remodeling GM, enhancing SCFA production, and restoring intestinal barrier function, thereby conferring protection via the gut–lung axis? To address these knowledge gaps, the present study was designed to evaluate the therapeutic efficacy and mechanistic basis of KBD in a murine model of IFV-induced severe pneumonia, with particular emphasis on GM and microbiota-derived metabolites. Our findings will contribute to a more precise understanding and more rational application of the strategy of KBD treatment on influenza.

## 2. Results

### 2.1. Chemical Analysis of KBD Extracts

**Figure 1:**
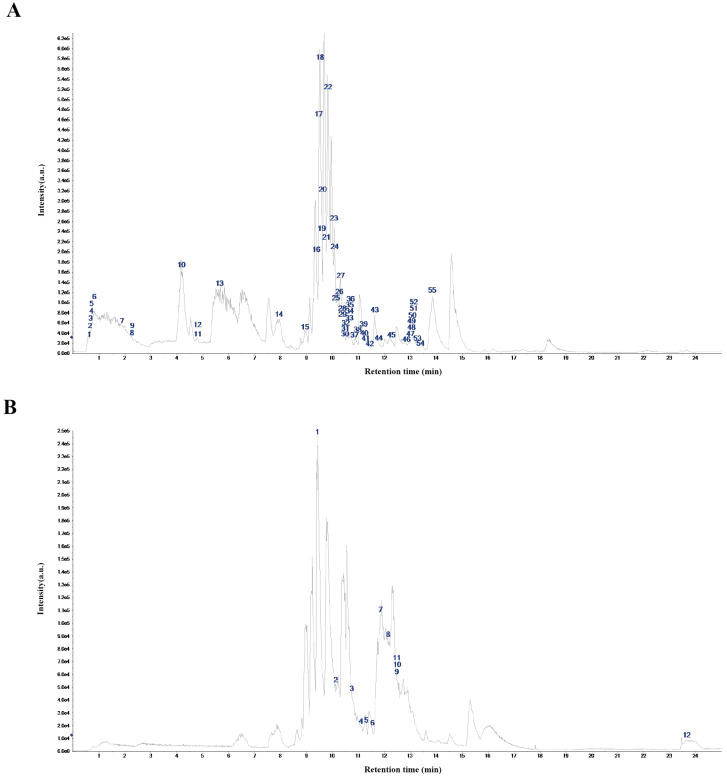
UPLC-QTOF-MS fingerprint analysis of KBD: (**A**) in positive ionization mode; (**B**) in negative ionization mode.

Chemical characterization of KBD granules was carried out by UPLC-Q-TOF-MS, yielding representative chromatographic fingerprints in both positive and negative ionization modes (Figure 1A,B). A total of sixty-seven distinct compounds were identified from the KBD formulation (Supplementary Tables S1 and S2). Major chemical classes comprised flavonoids, terpenoids, amino acids, lignans, alkaloids, and saponins. Notably, several of the identified components have documented bioactivities relevant to the study’s findings.

### 2.2. KBD Alleviated Lung Injury in the Mice with Severe Pneumonia Infected by Influenza Virus

**Figure 2:**
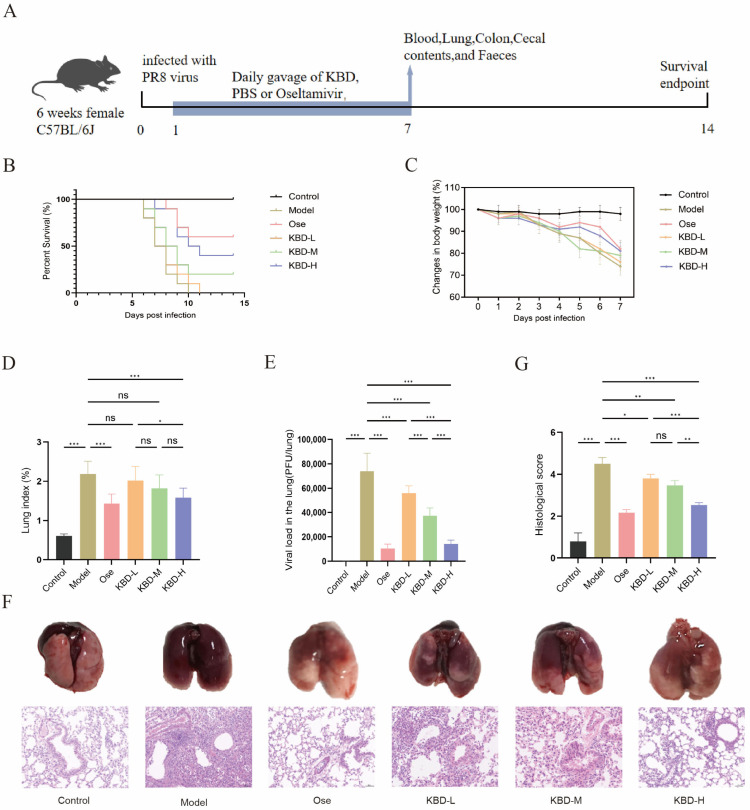
Protective effects of KBD in influenza virus-induced severe pneumonia mice. (**A**) Experimental timeline. (**B**) Body weight changes. (**C**) Survival curves. (**D**) Lung index. *n* = 10 (**E**) Pulmonary viral load, *n* = 5. (**F**) Representative lung photographs and H&E-stained lung sections (original magnification: 200×; scale bar: 50 µm). (**G**) Lung histopathological score, *n* = 3. Data in panels (**D**–**F**) are presented as mean ± standard deviation (SD). * *p* < 0.05, ** *p* < 0.01, *** *p* < 0.001; ns, not significant.

As outlined in the experimental timeline (Figure 2A), KBD treatment attenuated body weight loss in influenza virus-induced severe pneumonia mice compared with the model group (Figure 2B). Under lethal viral challenge, the survival rate at day 14 was 40% in the KBD-H group, which was higher than that in the KBD-M and KBD-L groups. The oseltamivir (Ose)-treated group showed 60% survival, whereas all mice in the model group died by day 10 (Figure 2C). KBD also reduced both the lung index and pulmonary viral load in a dose-dependent manner (Figure 2D,E). On day 7 post-infection, the viral titer in influenza virus-induced severe pneumonia mice was 74,080.47 ± 14,668.29 PFU/lung; KBD-H treatment significantly reduced the viral load to 14,166.62 ± 3121.47 PFU/lung. Gross examination showed dark red lung tissue with areas of consolidation in influenza virus-induced severe pneumonia mice, in contrast to the normal pink appearance in the control group. Lungs from both KBD- and oseltamivir-treated groups exhibited intermediate pathology with only partial consolidation (Figure 2F). Histopathological evaluation (H&E staining) revealed severe alveolar structural damage, marked thickening of alveolar walls, and extensive inflammatory cell infiltration and exudation in the model group. These pathological changes were notably alleviated by treatment with KBD or oseltamivir (Figure 2F,G).

### 2.3. KBD Alleviates Influenza-Induced Pulmonary Inflammation

**Figure 3:**
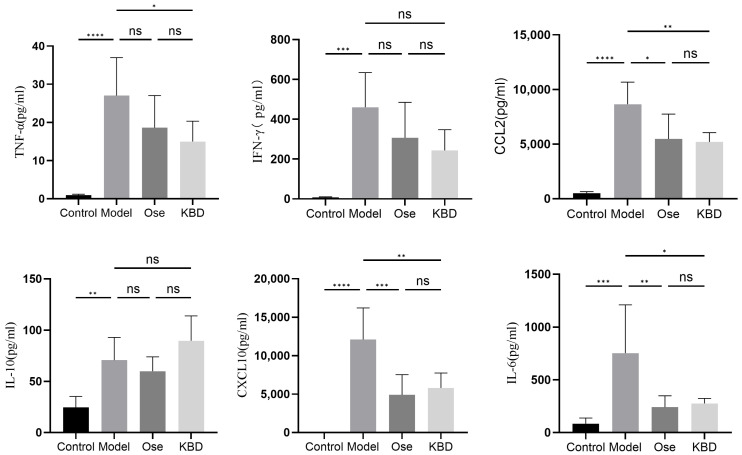
Effects of KBD on the levels of pro-inflammatory cytokines and chemokines in the lungs of influenza virus-induced severe pneumonia mice, *n* = 6. Data in panels are presented as mean ± standard deviation (SD). * *p* < 0.05, ** *p* < 0.01, *** *p* < 0.001, **** *p* < 0.0001; ns, not significant.

Based on these findings, we further evaluated the effects of the human-equivalent dose of KBD (KBD-H) on pulmonary inflammation in influenza virus-induced severe pneumonia mice. Infection with IFV significantly elevated the pulmonary levels of pro-inflammatory cytokines and chemokines, including TNF-α, IL-6, CXCL10, and CCL2, compared with the control group. Treatment with KBD or oseltamivir (Ose) effectively reduced the expression of these inflammatory mediators. Notably, the suppressive effects of both KBD and oseltamivir reached statistical significance compared to the model group (Figure 3).

### 2.4. KBD Ameliorates Intestinal Barrier Injury in Influenza Virus-Induced Severe Pneumonia Mice

**Figure 4:**
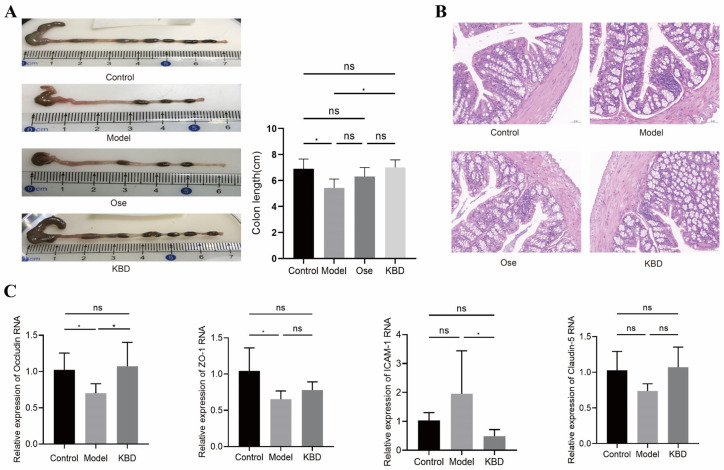
KBD alleviates intestinal inflammation and enhances barrier function in influenza virus-induced severe pneumonia mice. (**A**) Representative colon images and quantitative measurement of colon length. (**B**) Representative H&E-stained colon sections (original magnification, 200×; scale bar, 50 µm). (**C**) Relative mRNA expression levels of intestinal barrier markers (Occludin, ZO-1, ICAM-1, and Claudin-5) in colon tissue. Actb was used as the reference gene for RT-qPCR analysis, *n* = 5. Data in panels (**A**,**C**) are presented as mean ± standard deviation (SD). * *p* < 0.05; ns, not significant.

KBD treatment significantly restored the infection-induced shortening of colon length compared to the model group (Figure 4A). Histopathological examination by H&E staining revealed distinct and intact goblet cells across all groups. Notably, the model group exhibited large inflammatory cell foci (0–2 foci per section) infiltrating the colon tissue. In contrast, only occasional and smaller inflammatory foci were observed in the oseltamivir- and KBD-treated groups, whereas no evident inflammatory infiltration was detected in the control group (Figure 4B). At the molecular level, IFV infection significantly down-regulated the colonic mRNA expression of tight junction proteins Occludin, ZO-1, and Claudin-5, while up-regulating the expression of Intercellular Adhesion Molecule-1 (ICAM-1). Treatment with KBD effectively reversed these alterations, normalizing the expression of key intestinal barrier-related markers (Figure 4C). Collectively, these results indicate that KBD provides substantial protection to the intestinal barrier and attenuates local inflammation, suggesting that its therapeutic efficacy against influenza may involve the modulation of gut-associated pathways.

### 2.5. KBD Treatment Altered the Expressions of the Proinflammatory Genes and the SCFA Metabolism-Related Genes

**Figure 5:**
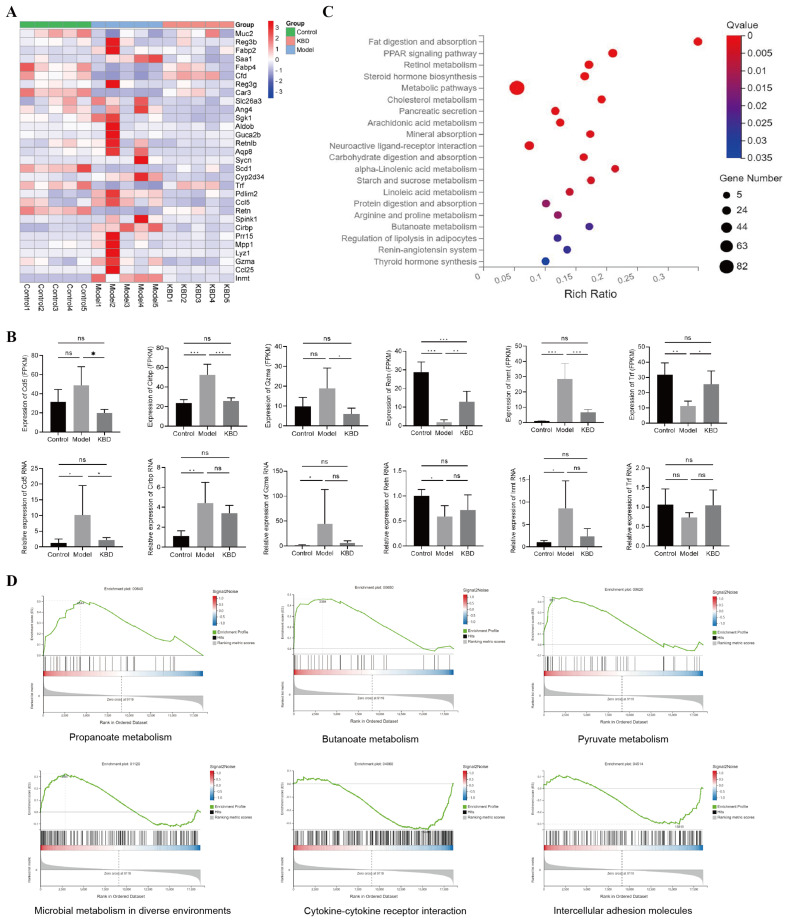
Colonic transcriptomics suggests KBD alleviates influenza virus-induced severe pneumonia by modulating gut microbiota and associated metabolites. (**A**) Heatmap of highly expressed DEGs between the KBD and model groups. Each row represents a gene, each column a sample. Color intensity reflects z-score normalized FPKM expression levels. (**B**) Validation of core DEG expression using FPKM values from RNA-seq and RT-qPCR, *n* = 5. (**C**) Top 20 enriched KEGG pathways for colonic DEGs, ranked by adjusted *p*-value. The *x*-axis indicates the enrichment score, bubble size represents the number of genes, and color corresponds to the significance level. (**D**) GSEA plots illustrating pathways up-regulated (green curves) by KBD treatment. Data in panels (**B**) are presented as mean ± standard deviation (SD). * *p* < 0.05, ** *p* < 0.01, *** *p* < 0.001; ns, not significant.

A total of 734 differentially expressed genes (DEGs) were identified between the KBD and model groups, with 154 up-regulated and 580 down-regulated. A heatmap of 30 highly expressed DEGs, based on z-score normalized FPKM values, is shown in Figure 5A. From these, six core DEGs were selected for further validation based on their reported roles in inflammation and lung injury: Ccl5, Cirbp, Gzma, and Inmt have been associated with exacerbated inflammatory responses when overexpressed [19,20,21], whereas Retn and Trf are likely related to relief of oxidative stress [22]. RT-qPCR analysis confirmed that KBD treatment significantly down-regulated the colonic expression of Ccl5, Cirbp, Gzma, and Inmt, while up-regulating Retn and Trf in influenza virus-induced severe pneumonia mice. These alterations were consistent with the FPKM profiles obtained from RNA sequencing (Figure 5B). KEGG analysis revealed that the DEGs were mainly enriched in multiple key pathways, including PPAR signaling, arachidonic acid metabolism, butanoate metabolism, cytokine–cytokine receptor interaction, and inflammatory mediator regulation of TRP channels (Figure 5C). Furthermore, GSEA indicated that KBD up-regulates pathways related to pyruvate metabolism, propanoate metabolism, butanoate metabolism, and microbial metabolism in diverse environments (Figure 5D).

### 2.6. KBD Restores Influenza-Induced Gut Microbiota Dysbiosis

**Figure 6:**
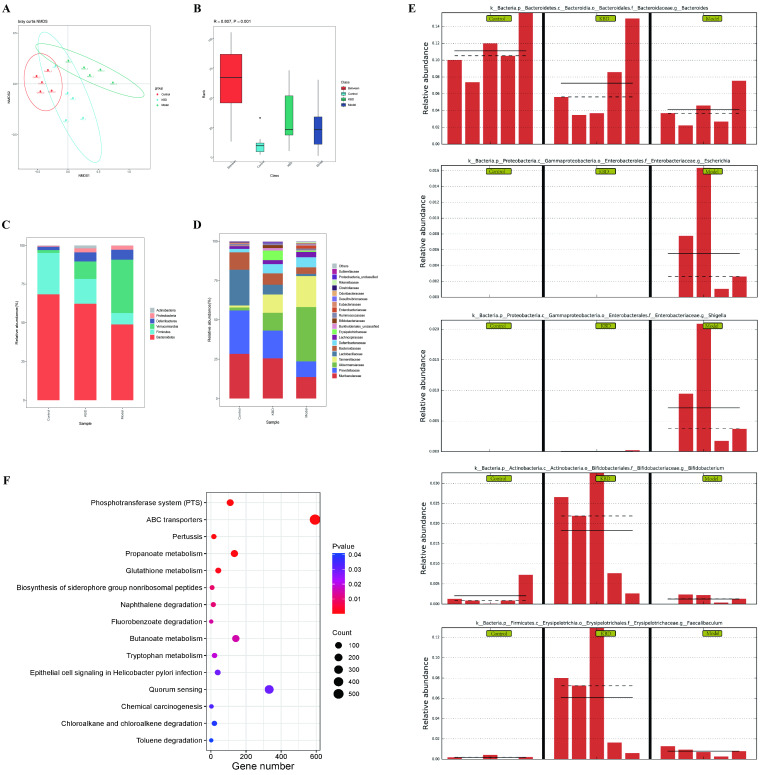
KBD modulates the gut microbiota in influenza virus-induced severe pneumonia mice. (**A**) NMDS analysis based on metagenomic data. (**B**) ANOSIS box plot illustrating differences in microbial community structure among groups. (**C**–**E**) Relative abundances of microbial taxa at the phylum (**C**), family (**D**), and genus (**E**) levels. (**F**) KEGG pathway enrichment analysis of differentially abundant genes between the KBD and model groups. The *x*-axis and dot size represent the number of genes enriched in each pathway; the *y*-axis lists pathway names, and dot color indicates the *p*-value.

Metagenomic sequencing was performed on fecal samples from control, PBS, and KBD groups. Samples underwent metagenomic sequencing. Non-metric multidimensional scaling (NMDS) revealed that the microbial communities of KBD-treated mice clustered closer to those of the control group than to the model group (Figure 6A). This structural shift was confirmed by ANOSIM, which indicated significant inter-group differences (R = 0.752, *p* = 0.001; Figure 6B). At the phylum level, KBD administration reduced the relative abundance of *Verrucomicrobia* and increased that of *Firmicutes* and *Bacteroidetes* in infected mice, thereby restoring the overall community structure toward a state resembling the control group (Figure 6C). At the family level, influenza infection decreased the abundances of *Bacteroidaceae*, *Lactobacillaceae*, *Prevotellaceae*, and *Muribaculaceae*, while increasing *Tannerellaceae* and *Akkermansiaceae*. These alterations were effectively reversed by KBD treatment (Figure 6D). Genus-level analysis further showed that infection reduced *Bacteroides* but increased *Escherichia* and *Shigella*; KBD restored these genera to near-normal levels. In addition, KBD significantly enhanced the abundance of beneficial genera such as *Bifidobacterium* and *Faecalibaculum*, which remained low in both control and model groups (Figure 6E). Functional analysis revealed that differential genes identified between groups were enriched in multiple metabolic pathways, including propanoate metabolism, butanoate metabolism, pyruvate metabolism, and tryptophan metabolism (Figure 6F).

Collectively, these findings demonstrate that KBD not only confers pulmonary and intestinal protection but also remodels the gut microbiota by restoring influenza-induced dysbiosis and enriching beneficial bacteria associated with metabolite production. These results support the hypothesis that the therapeutic effects of KBD are mediated, at least in part, through the modulation of gut microbial composition and its metabolic functions.

### 2.7. FMT from KBD-Treated Mice Alleviates Pulmonary and Intestinal Injury in IFV-Induced Severe Pneumonia Mice

**Figure 7:**
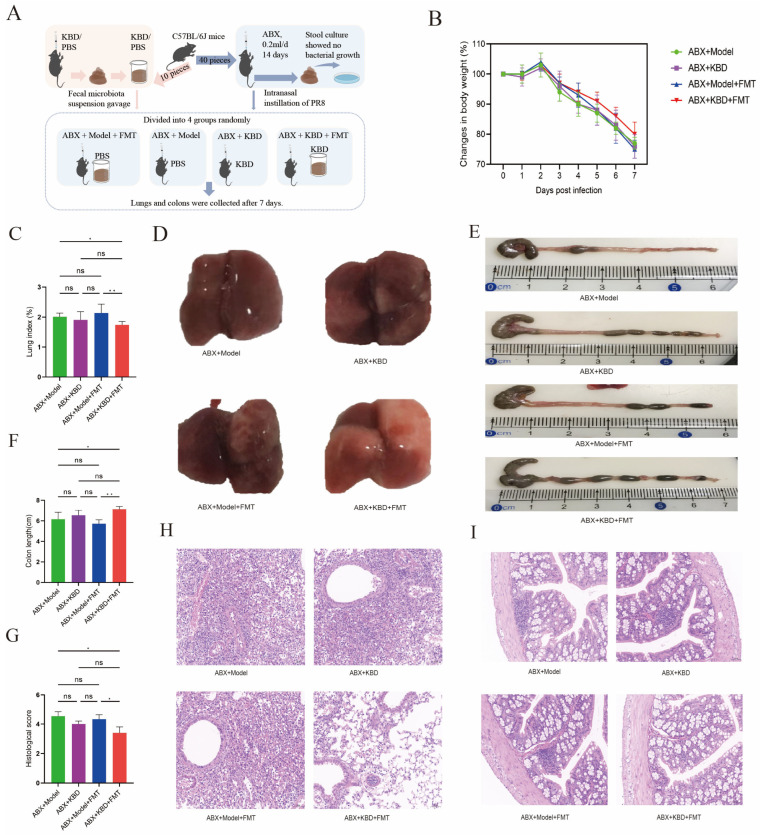
KBD-derived FMT confers pulmonary and intestinal protection in influenza virus-induced severe pneumonia mice. (**A**) Schematic diagram of the FMT experimental procedure. (**B**) Body weight changes. (**C**) Lung index, *n* = 10. (**D**) Representative lung photographs. (**E**) Representative colon photographs. (**F**) Colon length, *n* = 5. (**G**) Lung histopathological score, *n* = 3. (**H**) H&E-stained lung sections (200×; scale bar, 50 µm). (**I**) H&E-stained colon sections (200×; scale bar, 50 µm). Data in panels (**C**,**F**,**G**) are presented as mean ± standard deviation (SD). * *p* < 0.05, ** *p* < 0.01; ns, not significant.

To investigate whether KBD exerts its protective effects via GM modulation, we conducted FMT from KBD-treated donors (KBD+FMT) to antibiotic-pretreated recipient mice (Figure 7A). The results showed that KBD+FMT significantly ameliorated weight loss (Figure 7B) in GM-disrupted mice. Furthermore, KBD+FMT markedly reduced the lung index and attenuated pathological lung injury (Figure 7C,D), while also restoring the infection-induced shortening of colon length (Figure 7E,F). In contrast, direct oral administration of KBD to microbiota-depleted mice showed no significant effects on lung index or colon length (*p* > 0.05).

Histological examination via H&E staining revealed severe pulmonary damage in the Model, KBD, and Model+FMT groups of microbiota-depleted mice, characterized by disrupted alveolar architecture, thickened alveolar walls, substantial exudation, and extensive inflammatory infiltration. KBD+FMT treatment significantly alleviated these pathological alterations, resulting in well-preserved alveolar structure and diminished inflammatory cell infiltration (Figure 7G,H). Concurrently, KBD-FMT also ameliorated intestinal inflammatory injury (Figure 7I). These findings collectively indicate that the pulmonary and intestinal protection conferred by KBD in influenza virus-induced severe pneumonia mice is partly dependent on the functional remodeling of the gut microbiota.

### 2.8. KBD Restores SCFA Production in the Cecum of IFV-Induced Severe Pneumonia Mice

**Figure 8:**
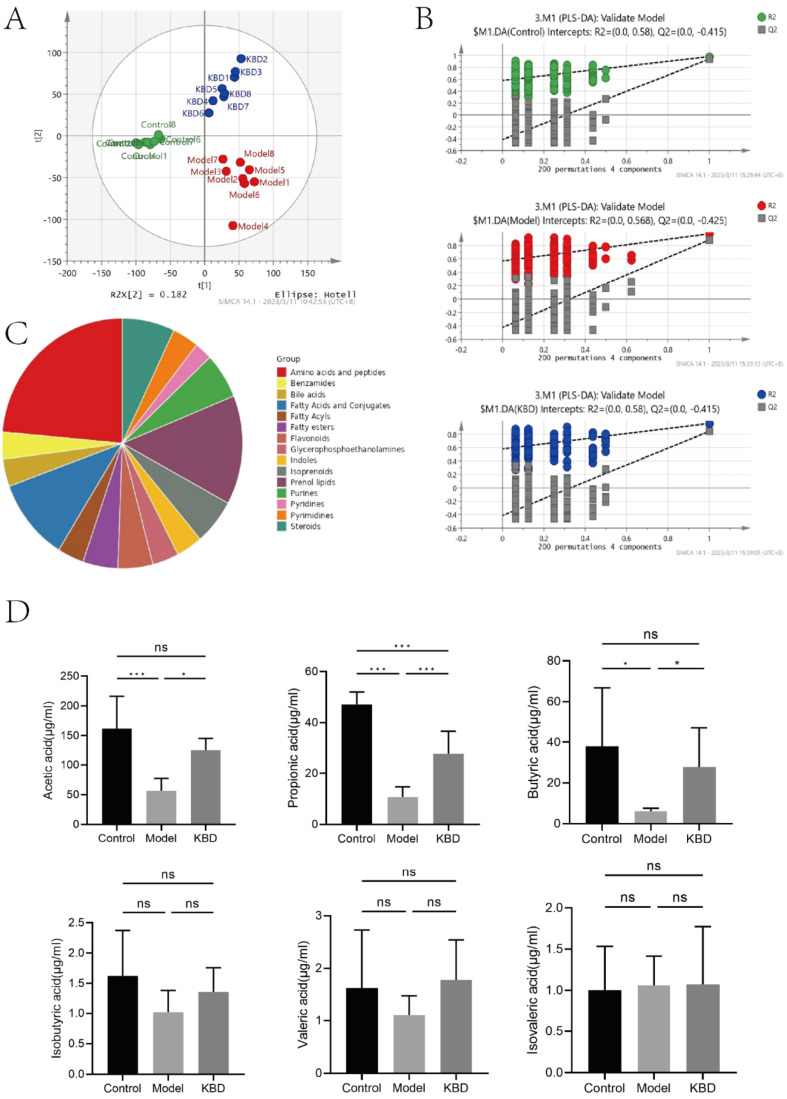
KBD intervention increases cecal SCFA levels in influenza virus-induced severe pneumonia mice. (**A**) PCA score plot from untargeted metabolomics of cecal contents. (**B**) Permutation test plot validating model stability (R_2_ > 0, Q_2_ < 0). (**C**) Classification pie chart of differentially abundant metabolites between the model and KBD groups. (**D**) Concentrations of major SCFAs in cecal contents across experimental groups, *n* = 8. Data in panels (**D**) are presented as mean ± standard deviation (SD). * *p* < 0.05, *** *p* < 0.001; ns, not significant.

To characterize the metabolic alterations induced by KBD intervention, cecal contents from mice were subjected to both untargeted metabolomics and targeted SCFA analysis. Principal component analysis of the untargeted metabolomics data showed clear separation between groups (Figure 8A), and permutation testing confirmed the stability and reliability of the established model (Figure 8B). A total of 320 differentially abundant metabolites (VIP > 1, *p* < 0.05) were identified between the model and KBD groups. These metabolites were primarily classified into three major categories: amino acids and peptides, propanoyl-containing lipids, and fatty acids with conjugates (Figure 8C).

Given the prior enrichment of SCFA-related pathways in both transcriptomic (GSEA) and metagenomic (KEGG) analyses—along with the detection of SCFA-precursor metabolites such as amino acids and dipeptides in the current untargeted metabolomics—we hypothesized that SCFAs are key mediators of KBD’s anti-influenza virus-induced severe pneumonia activity. Targeted quantification confirmed this hypothesis: the levels of acetic acid, propionic acid, and butyric acid were significantly decreased in the model group compared to the control group (*p* < 0.05), and KBD treatment effectively restored their concentrations (*p* < 0.05; Figure 8D).

## 3. Discussion

KBD, a classical TCM formula, was used clinically for influenza virus-induced severe pneumonia mice (example ARDS) for approximately 30 years [9,10,11]. In this study, we provide experimental evidence that KBD confers robust protection against IFV-infected pneumonia in mice. It markedly improved survival, alleviated lung pathological injury, and reduced the levels of inflammatory cytokines. Consistent with prior reports on TCM decoctions against influenza, KBD exhibited multi-faceted activities, including suppression of viral replication, attenuation of excessive inflammatory responses, and restoration of gut microbiota homeostasis.

To evaluate the efficacy of KBD treatment against severe and critical IFV-induced pneumonia, we established a severe pneumonia mouse model by increasing the IFV challenge dose from 360 PFU to 540 PFU. This higher viral load induced more severe pulmonary inflammatory injury (average lung index ~2.2) compared to our previous studies (Supplementary Figure S2), where the lung index typically ranged from 1.1 to 1.8 [23,24,25]. KBD treatment substantially attenuated the inflammatory response and reduced the lung index by 27.85% (from 2.19% to 1.58%), demonstrating superior performance compared to previously reported TCM formulas.

In 14-day survival assays, we further increased the lethal dose of IFV from 720 PFU to 1080 PFU. While this heightened challenge led to a lower overall survival as expected, KBD treatment significantly improved survival from 0% to 40% under these stringent conditions. Although this survival rate was lower than that of the oseltamivir group (60%), it is comparable to the efficacy of other TCM formulas tested against significantly lower lethal doses in prior studies [26,27,28,29,30].

A distinctive mechanistic feature of KBD was its robust capacity to improve gut microbiota. As expected, KBD reduced the abundance of potentially harmful bacteria such as *Escherichia* and *Shigella*, while restoring multiple beneficial taxa, including *Firmicutes*, *Bacteroidetes* and *Actinobacteria*. Notably, the relative abundance of *Firmicutes* and *Actinobacteria* in the KBD group even exceeded those of the non-infected group. Several genera of *Firmicutes* are major producers of butyric acid [31,32], whereas *Bifidobacterium* (*Actinobacteria*) is a principal producer of acetic acid and propionic acid [18,33,34,35]. Host-level evidence from colon transcriptomics further supported this functional shift: compared with PBS controls, IFV-infected mice receiving KBD showed up-regulation of pathways related to propanoate and butanoate metabolism. Consistently, targeted metabolomics of cecal contents demonstrated significantly higher levels of propionic acid and butyric acid in the KBD group than in the mock group. Collectively, these concordant findings across multi-omics layers indicate that KBD increases SCFA concentrations, likely mediated by enrichment of SCFA-producing probiotics.

SCFAs—particularly butyric acid—have been widely reported to restrain excessive inflammation. Mechanistically, it has been proposed that SCFAs can signal through receptors such as FFAR2 and promote regulatory T-cell (Treg) differentiation, including increased FOXP3 expression, thereby expanding Treg abundance and strengthening immunoregulatory capacity [36,37,38].

To directly evaluate the role of the gut microbiota in KBD-mediated protection against influenza, we performed fecal microbiota transplantation (FMT) experiments. Antibiotic (ABX)-mediated depletion of the gut microbiota markedly attenuated the effects of KBD. In the absence of intestinal flora, KBD alone produced little improvement in lung pathological injury in IFV-infected mice. However, when the intestinal microbiota was re-established by FMT, these microbiota-depleted mice regained the therapeutic benefit of KBD. Together, these results support a critical role for the intestinal microbiota in mediating KBD efficacy against influenza.

In addition to suppressing excessive inflammation, KBD also reduced influenza viral replication. After 7 days of treatment, KBD lowered the viral load by 80.88% (from 74,080.47 ± 14,668.29 PFU to 14,166.62 ± 3121.47 PFU), although the reduction was slightly smaller than that of oseltamivir (85.88%, from 74,080.47 ± 14,668.29 PFU to 10,456.99 ± 3561.00 PFU). Notably, unlike many previously reported anti-influenza TCM formulas, KBD does not contain the herbs commonly associated with direct anti-influenza activity, e.g., *Scutellariae Radix* (*Huangqin*; *Scutellaria baicalensis* Georgi), *Lonicerae Japonicae Flos* (*Jinyinhua*; *Lonicera japonica* Thunb.), *Houttuyniae Herba* (*Yuxingcao*; *Houttuynia cordata* Thunb.), *Bupleuri Radix* (*Chaihu*; *Bupleurum chinense* DC./*B. scorzonerifolium* Willd.), *Forsythiae Fructus* (*Lianqiao*; *Forsythia suspensa* (Thunb.) *Vahl*), and *Isatidis Radix* (*Banlangen*; *Isatis indigotica* Fort.). We therefore suppose that KBD may inhibit viral replication indirectly via enrichment of beneficial gut microbiota. This was based on the following reasons. We identified abundant flavonoids in KBD with UPLC/MS analyses in this study. These compounds may be metabolized into DAT by many beneficial gut microbiota such as *Lactobacillus, Bacteroidetes* and *Bifidobacterium*, and induced type I interferon responses and thereby suppress influenza virus proliferation [39,40].

Another notable characteristic of KBD treatment in IFV-infected mice was the apparent improvement in energy metabolism. We observed significant up-regulation of the pyruvate metabolism pathway following KBD administration, suggesting that pyruvate-linked tricarboxylic acid (TCA) cycling and mitochondrial oxidative phosphorylation may be enhanced. In this study, the mice were challenged with a higher dose of IFV and suffered more severe pulmonary infection. They likely experienced hypoxia due to pronounced ventilatory limitation and diffusion impairment (average lung index ~2.2). Hypoxia can impair cellular energy metabolism and promote excessive mitochondrial reactive oxygen species (ROS) production. Accumulated ROS can damage mitochondria and further compromise energy supply in cells and tissues in turn [41,42]. Energy failure in cells and inflammation storm may cause dysfunction of multiple organs, and excite greater harm than viral proliferation in hosts with severe or critical pneumonia.

From a TCM perspective, the primary therapeutic objective for severe IFV pneumonia is to ‘reinforce vital qi to dispel pathogens.’ In contemporary biological terms, this concept emphasizes prioritizing the restoration of host resistance and functional reserve alongside pathogen clearance, particularly in severe or critical cases. The core components of KBD are *Ginseng Radix et Rhizoma (Renshen)* and *Corni Fructus* (*Shanzhuyu*). *Panax ginseng* (*Renshen*) contains major bioactive ginsenosides (e.g., Rg1, Rb1, and Re), which have been reported to enhance oxidative phosphorylation by up-regulating mitochondrial respiratory complex activity and maintaining mitochondrial membrane potential. Furthermore, these ginsenosides improve mitochondrial biogenesis via the AMPK–PGC-1α signaling axis [43] and promote the expression of antioxidant enzymes. Complementing this, *Cornus officinalis* (*Shanzhuyu*) contains iridoid glycosides (e.g., sweroside and Cornin), which facilitate the clearance of damaged mitochondria by promoting mitophagy via the PINK1/Parkin pathway. These glycosides also mitigate mitochondrial injury by scavenging excessive reactive oxygen species (ROS) also through the up-regulation of antioxidant enzymes [44,45]. Consistent with these mechanisms, our UPLC/MS analysis confirmed a high abundance of multiple ginsenosides and sweroside within the KBD decoction.

Multiple TCM formulas have been reported to show therapeutic efficacy against influenza infection, such as *San-Yang-He-Zhi decoction*, *Ku-Kan granule*, *Qing-Fei-Ying decoction*, *Qing-Jin-Hua-Tan decoction*, *Hao-Qin-Qing-Dan decoction*, *Fei-Yan-Qing-Hua decoction*, and *Lian-Hua-Qin-Wen granule* [23,24,25,46,47,48,49]. Most of these formulas are derived from *Ma-Xing-Gan-Shi decoction* (a classical prescription from Treatise on Febrile Diseases) and typically include one to three herbs with reported direct antiviral activity (e.g., *Scutellariae Radix*, *Lonicerae Japonicae Flos*, *Houttuyniae Herba*, *Bupleuri Radix*, *Forsythiae Fructus*, and *Isatidis Radix*). These formulations largely emphasize symptom relief and pathogen elimination. Although KBD is also based on Ma-Xing-Gan-Shi decoction, it has been primarily used for severe pulmonary infection (e.g., ARDS), where the urgent therapeutic priority is to enhance host resistance and tolerance to severe infection. In alignment with this clinical orientation, KBD supplements *Ginseng Radix et Rhizoma* and *Corni Fructus* rather than the above-mentioned “virus-suppressing” herbs, which represents a key distinction from most previously reported anti-influenza TCM decoctions.

Despite our multi-layered mechanistic investigation, several limitations should be acknowledged. First, although FMT experiments demonstrated that gut microbiota depletion markedly attenuates KBD efficacy, direct supplementation of SCFAs in microbiota-depleted, IFV-infected mice would more definitively establish the causal contribution of SCFAs to KBD-mediated protection. Second, the inclusion of additional anti-influenza TCM formulas as comparative controls would allow for a more comprehensive assessment of KBD’s efficacy in treating IFV-induced severe pneumonia relative to existing therapies. Third, although multi-omics analyses suggested improved mitochondrial energy metabolism after KBD treatment (e.g., up-regulated pyruvate metabolism), we did not directly measure mitochondrial function or hypoxia-related metabolic resilience at the organ level; future studies should include functional assays (e.g., ATP production, oxidative phosphorylation/respiration, ROS, and organ injury markers) to verify whether KBD-driven metabolic improvement enhances tissue and organ resistance and tolerance to severe infection and hypoxic stress. Finally, the dose-conversion method used in this study was originally developed primarily for small-molecule drugs and may therefore have certain limitations when applied to complex traditional Chinese medicine extracts with multiple constituents and incompletely characterized bioavailability. Although such dose-conversion approaches have been widely used in studies of herbal medicines, caution is warranted when extrapolating findings from animal experiments to humans.

## 4. Materials and Methods

### 4.1. Ethics Statement

The animal study protocol was reviewed and approved by the Animal Welfare and Ethics Committee of the Chinese Center for Disease Control and Prevention (No. SYXK [Beijing] 2020-057, Beijing, China) on 10 March 2020.

### 4.2. Virus Strain

Influenza A virus A/Puerto Rico/8/34 (PR8) was provided by the laboratory of the National Institute for Communicable Disease Control and Prevention, Chinese Center for Disease Control and Prevention. The concentration of the viral stock was previously determined to be 4.5 × 10^6^ PFU/mL via plaque assay [50].

### 4.3. KBD Preparation and Quality Control

The composition and dosage of each herbal ingredient in the KBD formulation are listed in Table 1. All herbal materials met the quality standards specified in the Pharmacopoeia of the People’s Republic of China (2010 edition). Instead of raw herbs, a granule formulation prepared from a concentrated water extract by Beijing Tcmages Pharmaceutical Co., Ltd. (Beijing, China). was used in this study. KBD granules were prepared as described previously [23]. Briefly, a total of 280 g of herbal pieces was extracted and concentrated to yield 40 g of granules. Quality control was performed using UPLC-Q-TOF-MS under established analytical conditions [51]. Data processing and visualization utilized PeakView 2.2 software. Sixty-seven components were identified in the KBD granule samples (Supplementary Tables S1 and S2).

### 4.4. Animal Treatment

A total of sixty female C57BL/6J mice (4–5 weeks old, 13–15 g) were supplied by Beijing Vital River Laboratory Animal Technology Co., Ltd. (Beijing, China) (License No. SCXK [Beijing] 2021-0006). Animals were maintained under specific pathogen-free conditions at the Experiment Animal Center of the China Center for Disease Control and Prevention, with controlled temperature (25 °C), humidity (40%), and a 12 h light/dark cycle. After 7 days of acclimatization, the mice, with actual body weight of approximately 16–18 g, were randomly allocated into six groups (*n* = 10 per group): Control, Model, Oseltamivir control (19.19 mg/kg/day), KBD-L (1.3 g/kg/day), KBD-M (2.6 g/kg/day), and KBD-H (5.2 g/kg/day). The randomization sequence was generated by IBM SPSS Statistics 25.0 software using a computer-generated random number method.

All surgical and experimental procedures were approved by the Institutional Animal Care and Use Committee and conducted in accordance with relevant guidelines. For viral infection, mice were anesthetized by intraperitoneal injection of 1% sodium pentobarbital (40 mg/kg). Except for the Control group, which received 50 μL of phosphate-buffered saline (PBS) intranasally, all other mice were inoculated intranasally with 50 μL of viral suspension containing 1.08 × 10^4^ plaque-forming units (PFU)/mL. Drug treatments commenced 24 h post-infection and were administered daily via oral gavage for 6 consecutive days.

The high dose of KBD (KBD-H, 5.2 g/kg/day) was converted from the clinical human dose based on the formula described in previous studies [52,53]: Human dose: 40 g/day per 70 kg adult; mouse equivalent dose = 40 g/day × (9.1/70 kg) = 5.2 g/kg/day, where 9.1 represents the human-to-mouse equivalent dose ratio.

Oseltamivir phosphate capsules (75 mg per tablet; License No. 0221901015) supplied by Yichang East Sunshine Changjiang Pharmaceutical Co., Ltd. (Yichang, China) were used as the positive control. The Control and Model groups received an equivalent volume of PBS. Four hours after the final administration, mice were anesthetized with an intraperitoneal injection of 1% sodium pentobarbital and euthanized by cervical dislocation.

For survival analysis, a separate cohort of 60 mice (*n* = 10 per group) were infected with a lethal dose of 1080 PFU PR8 virus and treated as described above. Body weight and survival were monitored daily until day 14 post-infection, at which point all surviving animals were humanely euthanized.

### 4.5. Assessment of Acute Lung/Colon Injury and Pulmonary Viral Load

The bodyweight of mice was recorded daily.

After execution, mice lungs were surface-dried with filter paper and weighed. The Lung Index values were calculated as: Lung index = weight of lungs (g)/bodyweight (g) × 100%.

Lung and colon tissues were fixed in 4% paraformaldehyde solution (G1101, Servicebio, Wuhan, China) for 48–72 h, paraffin-embedded, sectioned at 5 µm, and stained with hematoxylin and eosin (H&E). Furthermore, we quantified the pathological changes in lung tissues using a scoring system, according to reference [54].

Lung tissues were homogenized using a tissue grinder (Tissuelyser II, QIAGEN, Düsseldorf, Germany) and subsequently centrifuged at 12,000 rpm for 10 min at 4 °C.

Viral RNA was extracted from the supernatant using the QIAamp Viral RNA Mini Kit (QIAGEN GmbH, Düsseldorf, Germany; Lot No. 163014099). Subsequently, influenza A viral RNA levels were quantified using an Influenza A Virus RT-qPCR Kit (Jiangsu Hechuang Biotechnology Co., Ltd., Zhenjiang, China; Lot No. 19100801) following the manufacturer’s protocol on an ABI 7500 Real-Time PCR System (Applied Biosystems, Carlsbad, CA, USA).

### 4.6. Measurement of Pulmonary Inflammatory Cytokines and Chemokines

A total of six cytokines and chemokines (TNF-α, IL-6, CCL2, CXCL10, IFN-γ, and IL-10) were quantified in lung tissue homogenates using the LabEx Biomarker Mouse 6-Plex Kit (R&D Systems, Minneapolis, MN, USA). The assay was performed on a Luminex 200 platform (Luminex, Austin, TX, USA) according to the manufacturer’s instructions.

### 4.7. Transcriptome Sequencing

Total RNA was isolated from colon tissues using TRIzol (Thermo Fisher Scientific, Waltham, MA, USA; Lot No. 15596018). RNA integrity was assessed using a Fragment Analyzer FSv2-CEAATI capillary electrophoresis system (Agilent Technologies, Santa Clara, CA, USA). High-quality RNA samples were used to construct sequencing libraries. Paired-end sequencing (150 bp) was performed on the BGISEQ-500 platform (BGI Genomics, Shenzhen, China). Raw reads underwent quality control and filtering using FASTQC. Clean reads were aligned to the reference genome using HISAT2. Gene expression levels were quantified as Fragments Per Kilobase of transcript per Million mapped reads (FPKM) using RSEM. Differential expression analysis identified Differentially Expressed Genes (DEGs), validated by RT-qPCR. Functional enrichment analyses (KEGG, Gene Set Enrichment Analysis—GSEA) were performed using clusterProfiler software.

### 4.8. Differentially Expressed Genes Being Validated by Real-Time Quantitative PCR (RT-qPCR)

Total RNA was extracted from mid-lung and mid-colon tissues. cDNA synthesis was performed using reverse transcription reagents. RT-qPCR reactions were prepared using specific primers (synthesized by Beijing Deaopin Biotechnology Co., Ltd., Beijing, China), template cDNA, and PCR master mix according to manufacturer protocols. Amplification and detection were carried out using a QuantGene9600 Real-Time PCR System (Hangzhou Bioer Technology Co., Ltd., Hangzhou, China). Gene expression levels were quantified using the 2^−ΔΔCT^ method for relative quantification.

### 4.9. Metagenome Analysis of Mouse Feces

As described in reference [23], fecal metagenomic DNA was extracted using the QIAamp DNA Stool Mini Kit (Qiagen, Germany, Düsseldorf, Germany). DNA concentration and purity were measured using a Qubit fluorometer with the Qubit dsDNA HS Assay Kit (Invitrogen, Carlsbad, CA, USA). DNA integrity was verified by 1% agarose gel electrophoresis. Qualified DNA samples were used to construct sequencing libraries with the NEBNext Ultra DNA Library Prep Kit for Illumina (NEB, Ipswich, MA, USA). Library quality and size distribution were assessed using an Agilent 2100 Bioanalyzer and quantified by qPCR. Paired-end sequencing (150 bp) was performed on the Illumina NovaSeq 6000 platform (Berry Genomics, Beijing, China). Raw reads were quality-trimmed using Trim Galore! and assessed with FastQC. De novo assembly was performed, and contig quality was evaluated using QUAST. Gene prediction was executed with MetaGeneMark. Non-redundant gene catalogs were generated using CD-HIT. Reads were mapped to the non-redundant gene catalog using Bowtie2 for abundance calculation. Functional annotation utilized KOBAS 3.0 for KEGG pathway assignment. Taxonomic profiling and relative abundance analysis were performed using MetaPhlAn2 by aligning reads to a database of ~17,000 microbial marker genes. Differential abundance analysis and KEGG enrichment of differentially abundant features between groups were conducted.

### 4.10. Metabolomic Analysis of Cecal Content and Quantification of SCFAs

Cecal content samples were thawed, homogenized, and subjected to ultrasonic extraction in a 4 °C water bath for 10 min, followed by centrifugation (25,000× *g*, 15 min). The supernatant was collected after two extraction cycles. Metabolite separation was achieved using a BEH C18 column (1.7 µm, 2.1 × 100 mm). Mobile phases consisted of: (A) water containing 0.1% formic acid and (B) 100% methanol containing 0.1% formic acid for positive ion mode; (A) water containing 10 mM ammonium formate and (B) 95% methanol containing 10 mM ammonium formate for negative ion mode. Mass spectrometric analysis was performed on a Q Exactive mass spectrometer (Thermo Fisher Scientific, Waltham, MA, USA) operating in full scan and data-dependent MS/MS acquisition modes.

Cecal content SCFA quantification was performed using a modified protocol based on reference [55]. Standard stock solutions of acetate, propionate, butyrate, isobutyrate, valerate, and isovalerate were diluted in diethyl ether. Analysis was conducted using a Thermo Scientific Trace 1300 gas chromatograph coupled to an ISQ 7000 single quadrupole mass spectrometer (Thermo Fisher Scientific, Waltham, MA, USA).

### 4.11. Fecal Microbiota Transplantation (FMT)

The FMT procedure was performed as previously described with modifications [55]. Recipient mice received an antibiotic cocktail (ampicillin 1 g/L, vancomycin 500 mg/L, gentamicin 500 mg/L) in drinking water ad libitum for 14 days to deplete gut microbiota. Successful depletion was confirmed by fecal culture showing no microbial growth. One day after antibiotic withdrawal, mice were infected with PR8 virus to establish the influenza virus-induced severe pneumonia model. Fresh fecal pellets (200 mg) collected from donor mice (Model or KBD-treated group) were suspended in 7.5 mL of sterile PBS, thoroughly vortexed, and filtered through a 70 µm cell strainer to remove particulate matter. Each recipient mouse received 200 µL of the resulting fecal suspension via oral gavage once daily for 6 consecutive days.

### 4.12. Data Analysis and Statistics

All statistical analyses were performed using IBM SPSS Statistics (Version 25.0) and GraphPad Prism (Version 9.5.0). Continuous data (e.g., body weight, lung index, colon length) are presented as mean ± standard deviation (SD) and were analyzed by one-way analysis of variance (ANOVA) or the non-parametric Kruskal–Wallis test, depending on whether data met assumptions of normality and homogeneity of variances. Post hoc comparisons among groups were conducted using Bonferroni or Least Significant Difference (LSD) tests, as appropriate. Survival data were analyzed by the log-rank (Mantel–Cox) test and visualized using Kaplan–Meier curves. For microbial community analysis, beta diversity was visualized using Principal Coordinates Analysis (PCoA), and group differences were assessed by non-parametric multivariate analysis of variance (PERMANOVA) based on the Adonis method. In transcriptomic analysis, differentially expressed genes (DEGs) were identified and subjected to KEGG pathway enrichment analysis using the clusterProfiler R package. Gene Set Enrichment Analysis (GSEA) was further employed to interpret expression patterns in the context of biological pathways and their directional trends. Metabolomic data processing was performed using Compound Discoverer 3.0. Unsupervised Principal Component Analysis (PCA) was used for initial dimensionality reduction and outlier detection, while supervised Partial Least Squares-Discriminant Analysis (PLS-DA) was applied to maximize group separation and validate model quality. A two-tailed *p*-value of less than 0.05 was considered statistically significant for all tests.

## 5. Conclusions

In conclusion, this study demonstrates that KBD confers significant protection against severe IFV-induced pneumonia in mice. Restoration of gut microbiota homeostasis and the resulting increase in SCFA production—thereby suppressing excessive inflammation—appear to be key drivers of KBD efficacy. In parallel, improved energy metabolism may enhance tissue and organ resistance and tolerance to severe infection and hypoxic stress, representing another important component of the protective mechanism.

## Figures and Tables

**Table 1:** Composition of KBD.

Chinese Name/Latin Name	English Name	Scientific Names	Amount (g)	Plant Part	Lot Number
Ma Huang/*Herba Ephedrae*	Ephedra	*Ephedra sinica* Stapf	9	Stem	18445879
Ku Xing Ren/*Semen Armeniacae Amarum*	Bitter apricot seed	*Prunus armeniaca* L.	9	Seed	19012331
Shi Gao/*Gypsum Fibrosum*	Gypsum	Gypsum (mineral)	30	Mineral (Gypsum)	20745642
Ren Shen/*Radix et Rhizoma Ginseng*	Ginseng	*Panax ginseng* C. A. Mey.	20	Root and Rhizome	18898769
Shan Zhu Yu/*Fructus Corni*	Cornelian Cherry	*Cornus officinalis* Siebold & Zucc.	30	Fruit	19567122
Bi Xie/*Rhizoma Dioscoreae Hypoglaucae*	Hypoglaucous Collett Yam	*Dioscorea hypoglauca* Palib.	10	Rhizome	20219843
Can Sha/*Faeces Bombycis*	Silkworm Droppings	*Bombyx mori* Linnaeus	12	Larval Droppings of Silkworm	18125644
Yi Yi Ren/*Semen Coicis*	Coix Seed	*Coix lacryma-jobi* L. var. ma-yuen (Rom.Caill.) Stapf	20	Seed	19874563
Zhu Ling/*Polyporus*	Chuling	*Polyporus umbellatus* (Pers.) Fries	20	Sclerotium of Umbrella Polypore Fungus	18435557
Tu Fu Ling/*Rhizoma Smilacis Glabrae*	Glabrous Greenbrier Rhizome	*Smilax glabra* Roxb.	30	Rhizome	19654987
Yu Jin/*Radix Curcumae*	Turmeric Root Tuber	*Curcuma longa* L.	10	Root Tuber	20112233
Chi Shao/*Radix Paeoniae Rubra*	Red Peony Root	*Paeonia lactiflora* Pall.	20	Root	19267502
Ban Xia/*Rhizoma Pinelliae*	Pinellia Tuber	*Pinellia ternata* (Thunb.) Breit.	9	Tuber	20764198
Gua Lou/*Fructus Trichosanthis*	Snakegourd Fruit	*Trichosanthes kirilowii* Maxim.	30	Fruit	19238671
Zhi Shi/*Fructus Aurantii Immaturus*	Immature Bitter Orange	*Citrus × aurantium* L.	15	Immature Fruit	20891036
Da Huang/*Radix et Rhizoma Rhei*	Rhubarb	*Rheum palmatum* L.	6	Root and Rhizome	19773284

## Data Availability

The original contributions presented in this study are included in the article/Supplementary Material. Further inquiries can be directed to the corresponding authors.

## Supplementary Materials

The following supporting information can be downloaded at: https://www.mdpi.com/article/10.3390/ph19071029/s1, Supplementary Table S1: Chemical Composition of Kai-Bi-Bu-Fei Decoction (in positive ionization mode); Supplementary Table S2: Chemical Composition of Kai-Bi-Bu-Fei Decoction (in negative ionization mode); Supplementary Figure S1: Percent Survival of mice infected with influenza virus under different infectious doses; Supplementary Figure S2: Lung index of mice infected with influenza virus under different challenge doses.
