# Supplementary material for: Kai-Bi-Bu-Fei Decoction Protects Mice Against Influenza Virus-Induced Severe Pneumonia via Gut Microbiota–Short Chain Fatty Acid Axis
Source: Pharmaceuticals (Basel). 2026 Jun 30;19(7):1029. doi: 10.3390/ph19071029 (PMC13414940; doi:10.3390/ph19071029)
Supplement: Supplementary file 1 [file pharmaceuticals-19-01029-s001.zip › pharmaceuticals-4323627-supplementary.pdf]

Supplementary File

Supplementary Table S1 Chemical Composition of Kai-Bi-Bu-Fei Decoction (in positive ionization mode)

No	Retention time	Formula	Adduct/ charge	Experimental mass	Identification
1	0.63	C6H14O6	[M + H]+	183.08632	Sorbitol
2	0.65	C6H14N4O2	[M + H]+	175.11896	L(+)-Arginine
3	0.67	C5H9NO4	[M + H]+	148.06044	Glutamic acid
4	0.72	C5H11NO2	[M + H]+	118.08626	Betaine
5	0.72	C5H9NO2	[M + H]+	116.07061	Proline
6	0.83	C7H13NO2	[M + H]+	144.10191	Stachydrine
7	1.89	C6H6N2O	[M + H]+	123.05529	Nicotinamide
8	2.28	C6H13NO2	[M + H]+	132.10191	Isoleucine
9	2.29	C17H14O7	[M + H]+	331.08122	Tricin
10	4.19	C10H13N5O4	[M + H]+	268.10402	Adenosine
11	4.82	C15H24N2O	[M + H]+	249.19613	Matrine
12	4.84	C10H13N5O5	[M + H]+	284.09895	Guanosine
13	5.65	C10H15NO	[M + H]+	166.12265	Pseudoephedrine
14	7.97	C11H12N2O2	[M + H]+	205.09715	L-Tryptophan
15	8.95	C17H24O10	[M + H]+	389. 14421	Cornin
16	9.38	C24H32O4. .HCOOH	[M + H]+	431.24281	Hecogenin
17	9.48	C16H22O9	[M + H]+	359.13365	Sweroside
18	9.53	C14H21O5N3	[M + H]+	312.15541	Leonurine
19	9.59	C23H28O11	[M + H]+	481.17042	Albiflorin
20	9.63	C21H28O13	[M + H]+	489.16026	Curculigoside + Na
21	9.77	C29H36O15	[M + H]+	625.21272	Neferine
22	9.83	C26H28O14	[M + H]+	565.15516	Schaftoside
23	10.07	C32H44N2O8. HCOOH	[M + H]+	631.32252	Solanesol
24	10.09	C27H30O14	[M + H]+	579.17085	Vitexin-2-O-rhamnoside
25	10.15	C20H19NO5	[M + H]+	354.13359	Protopine
26	10.27	C21H25NO4	[M + H]+	356.18562	Tetrahydropalmatine

27	10.31	C10H8O4	[M + H]+	193.04953	Isoscopoletin
28	10.39	C15H12O7	[M + H]+	305.06557	Dihydroquercetin
29	10.42	C21H22O9	[M + H]+	419.13365	Liquiritin
30	10.48	C27H32O14	[M + H]+	581.18648	Narirutin
31	10.51	C22H27NO4	[M + H]+	370.20128	Corydaline
32	10.54	C15H12O5	[M + H]+	273.07576	Naringenin
33	10.66	C28H34O15	[M + H]+	611.19703	Neohesperidin
34	10.67	C22H24O10	[M + H]+	449.14421	Quercetin 7-rhamnoside;Vincetoxicoside B
35	10.68	C15H10O7	[M + H]+	303.04994	Morin hydrate
36	10.71	C16H14O6	[M + H]+	303.08632	Hesperetin
37	10.85	C21H22O10	[M + H]+	435.12858	Engeletin
38	10.99	C18H29NO3	[M + H]+	308.22203	Glutathione
39	11.22	C27H34O11.NH3	[M + H]+	552.24391	Arctiin +NH3
40	11.24	C21H18O11	[M + H]+	447.09218	Apigenin 7-O-beta-D-glucuronide
41	11.32	C39H50O19	[M + H]+	823.30189	Epimedin C
42	11.45	C33H40O15	[M + H]+	677.24397	Icarrin
43	11.64	C15H10O4	[M + H]+	255.06518	Chrysophanol
44	11.8	C30H32O12.NH3	[M + H]+	602.22322	Benzoylpaeoniflorin +NH3
45	12.28	C30H48O5	[M + H]+	489.35747	Asiatic acid
46	12.86	C20H20O7	[M + H]+	373.12819	Isosinensetin
47	13.02	C15H18O2	[M + H]+	231.13795	Dehydrocostus lactone
48	13.04	C15H20O3	[M + H]+	249.14853	Parthenolide
49	13.04	C26H30O8	[M + H]+	471.20134	Limonin
50	13.08	C25H26O6	[M + H]+	423.18022	Sanggenol L
51	13.12	C13H18O7	[M + H]+	287.11253	Salicin
52	13.16	C15H24	[M + H]+	205.19508	Patchouli alcohol (loss H20)
53	13.29	C21H22O8	[M + H]+	403.13875	Nobiletin
54	13.37	C15H24O2	[M + H]+	237.1849	Curdione
55	13.87	C15H22O2	[M + H]+	235.16926	Curcumenol

Supplementary Table S2 Chemical Composition of Kai-Bi-Bu-Fei Decoction (in negative ionization mode)

No	Retention time	Formula	Adduct/ charge	Experimental mass	Identification
1	9.43	C20H22O8.HCOOH	[M-H]-	435.12966	Polydatin +HCOOH
2	10.13	C21H20O9	[M-H]-	415.10344	Daidzin
3	10.75	C27H30O16	[M-H]-	609.1461	Rutin
4	11.11	C33H44O17	[M-H]-	711.2506	(-)-Syringaresnol-4-O-β-D-apiofuranosyl-(1→2)-β-D-glucopyran
5	11.31	C30H45ClO6	[M-H]-	535.28319	Senegenin
6	11.54	C22H26N4	[M-H]-	345.20848	Corticosterone
7	11.85	C26H45NO6S.Na	[M-H]-	521.27928	20(S)-Protopanaxatriol +HCOOH
8	12.15	C41H68O14.HCOOH	[M-H]-	829.45909	Ginsenoside Rg2+HCOOH
9	12.49	C20H28O4	[M-H]-	331.19148	Dehydroandrographolide
10	12.5	C24H40O4	[M-H]-	391.28539	Deoxycholic acid
11	15.5	C20H30O	[M-H]-	285.2224	Kaempferol
12	23.66	C42H66O15	[M-H]-	809.43291	APedunsaponin A

Based on the foundational theories of Traditional Chinese Medicine (TCM), our team systematically delineated the disease progression following influenza virus infection and explored stage-specific TCM intervention strategies—targeting early influenza, influenza virus–induced pneumonia, and severe pneumonia—with representative decoctions including the San-Yang-He-Zhi (SYHZ) decoction[1],Kugan granules(KGKL)[2] and Qing-Fei-Yin decoction(QFY)[3].

To clarify the differing levels of infection severity addressed by these therapies, we summarized the viral challenge doses used in the aforementioned studies and presented the survival rates (Supplementary Fig. 1) and lung index (Supplementary Fig. 2) of their respective model groups.In the present study, we successfully established a mouse model of influenza virus-induced severe pneumonia for the first time using an infectious dose of 540 PFU. As the viral dose increased, the model group corresponding to KBD treatment showed the most severe outcome, with the shortest survival time and the greatest increase in lung index.

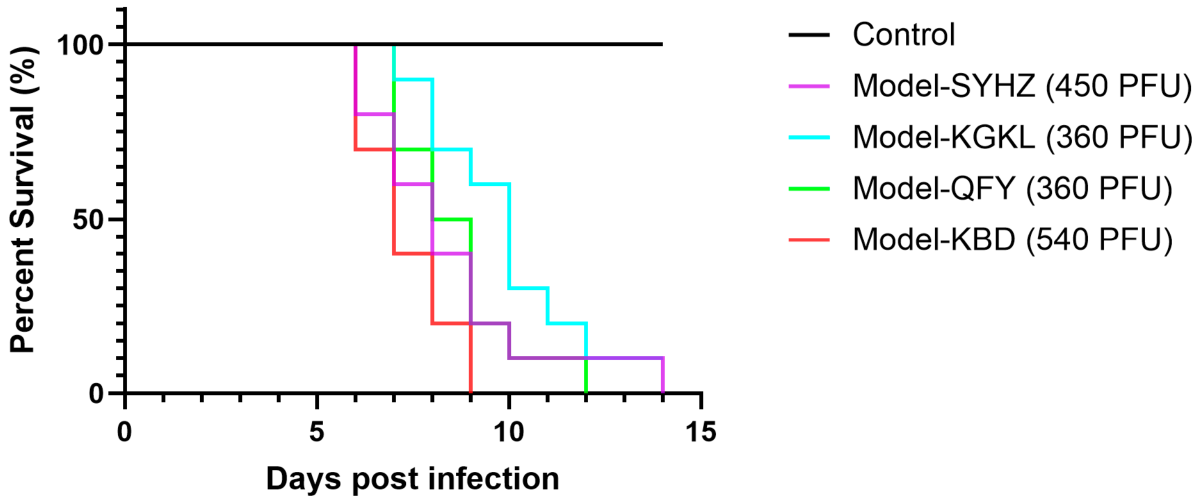

Supplementary Fig. S1 Percent Survival of mice infected with influenza virus under different infectious doses

Groups: Control; SYHZ (San-Yang-He-Zhi decoction, 450 PFU); KGKL (Kugan granules, 360 PFU); QFY (Qing-Fei-Yin decoction, 360 PFU); KBD (KaiBi-BuFei decoction, 540 PFU). With increasing viral doses, survival rates declined and mortality occurred earlier. Mice in the KBD model group (540 PFU) died earliest, indicating the most severe lung injury. All control mice survived through the observation period.

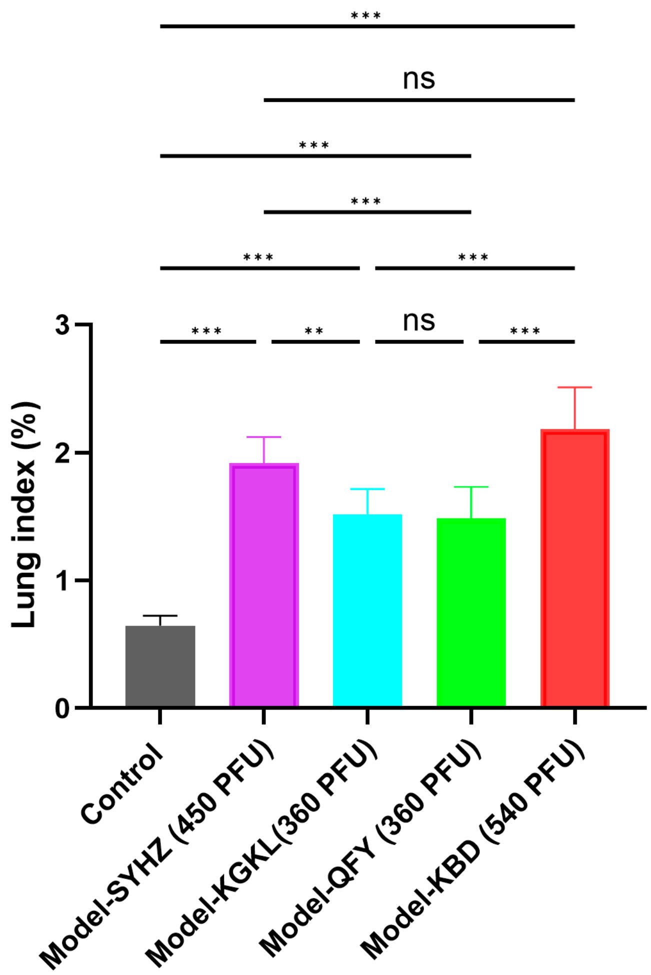

Supplementary Fig. S2 Lung index of mice infected with influenza virus under different challenge doses

Groups: Control; SYHZ (San-Yang-He-Zhi decoction, 450 PFU); KGKL (Kugan granules, 360 PFU); QFY (Qing-Fei-Yin decoction, 360 PFU); KBD (KaiBi-BuFei decoction, 540 PFU). The lung index showed a dose-dependent increase. The KBD model group (540 PFU) exhibited the highest lung index, confirming successful establishment of a severe pneumonia model at this dose.

References:

1. Cheng M, Zhang Y, Yan J, Huang Y, Wang M, Zhai Z, et al. Inhibiting virus replication and excessive inflammatory response: mechanism of combined prescription of ma-xing-shi-gan decoction and xiao-chai-hu decoction against influenza virus. J Ethnopharmacol. 2023;313:116481. https://doi.org/10.1016/j.jep.2023.116481

2. Ma H, He S, Yang T, Lu S, Wang X, Yue K, et al. Possible mechanism of Chinese patent Kugan granules against influenza infection: inducing interferon I and suppressing inflammation. Guidel Stand Chin Med. 2025;3:155. https://doi.org/10.1097/gscm.0000000000000058

3. Nie W, Wang M, Cheng M, Ren Z, Liu G, Liu C, et al. The mechanism of qing-fei-yin decoction against influenza: synergistical inhibition on viral replication and inflammation. J Ethnopharmacol. 2026;358:120891. https://doi.org/10.1016/j.jep.2025.120891
